# The Role of Negative Pressure Therapy in Diabetic Foot Ulcer: A Meta‐Analysis

**DOI:** 10.1111/1753-0407.70226

**Published:** 2026-04-08

**Authors:** Hai Du, Tao Jiang, Renqun Mao, Xiaofan Yang, Zhenbing Chen

**Affiliations:** ^1^ Department of Hand Surgery Union Hospital, Tongji Medical College, Huazhong University of Science and Technology Wuhan China; ^2^ Department of Hand and Foot Surgery Shenzhen Nanshan People's Hospital Shenzhen China; ^3^ Hubei Key Laboratory of Regenerative Medicine and Multi‐Disciplinary Translational Research (Huazhong University of Science and Technology) Wuhan China; ^4^ Hubei Provincial Clinical Research Center for Chronic Wound and Diabetic Foot Wuhan China

**Keywords:** amputation, diabetic foot ulcer, meta‐analysis, negative pressure wound therapy, wound healing rate

## Abstract

Diabetic foot ulcer (DFU) is a severe complication, often leading to amputation. Negative pressure wound therapy (NPWT) is an advanced treatment option requiring further consolidated evidence. This study evaluated the effectiveness and safety of NPWT versus conventional wound care for DFU, focusing on wound healing, amputation prevention, and adverse events (AEs). We systematically searched PubMed, Cochrane Library, Embase, and Web of Science for randomized controlled trials (RCTs) from inception until October 2025. Twenty‐three RCTs involving 2086 participants were included. Two reviewers independently selected studies. Outcomes included wound healing rate, ulcer area reduction, amputation rate, and adverse events. Pooled odds ratios (OR) and standardized mean differences (SMD) with 95% confidence intervals (CI) were calculated using random‐effects models. NPWT significantly improved the wound healing rate (OR 4.48, 95% CI 2.58–7.77), led to a greater reduction in ulcer area (SMD 1.26, 95% CI 0.70–1.82), and substantially lowered amputation risk (OR 0.35, 95% CI 0.19–0.64) compared to conventional care. No significant difference was found in adverse events (OR 0.98, 95% CI 0.56–1.71). Subgroup analyses confirmed benefits across ages, follow‐up durations, and control dressings. The healing benefit was most pronounced in less severe ulcers (Wagner grade < 2), while amputation protection was consistent across severities. NPWT is an effective and safe strategy for DFU, significantly accelerating wound healing and reducing amputation risk, supporting its integration as a cornerstone adjunctive therapy.

## Highlights

- NPWT significantly improves wound healing (OR 4.48) and reduces amputation risk (OR 0.35) in patients with diabetic foot ulcers.
- Subgroup analyses confirm consistent benefits across different ulcer severities, patient ages, and follow‐up durations.
- NPWT does not increase the incidence of adverse events compared to conventional wound care.

## Graphical abstract

Negative pressure wound therapy (NPWT) is an advanced treatment that applies controlled subatmospheric pressure to a wound dressing. This meta‐analysis of 23 RCTs demonstrates that NPWT significantly improves wound healing (OR = 4.48) and reduces amputation risk (OR = 0.35) in diabetic foot ulcer (DFU), without increasing adverse events.

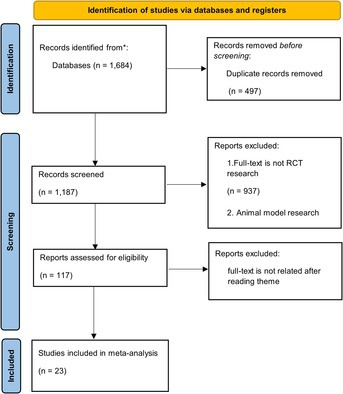

## 1 Introduction

Diabetic foot ulcer (DFU) is defined as a break in the skin of the foot in people with diabetes, extending at least through the epidermis and into part of the dermis [1]. It represents one of the most common and severe chronic complications of diabetes, characterized by foot infection and deep tissue injury resulting from vascular (particularly occlusion of medium and small arteries in the lower leg) and neuropathic pathologies. The incidence of DFU among diabetic patients is notably high, ranging from 19% to 34%, with approximately 20% of foot ulcers leading to amputations of varying degrees, posing a serious threat to patient quality of life and overall health [2].

Current standard management of DFU centers on core principles including wound debridement, revascularization, infection control, and off‐loading [3]. However, diabetic wound healing impairment involves multifactorial mechanisms, such as local microcirculatory dysfunction [4, 5, 6], dysfunction of wound cells [7, 8], and degeneration of small sensory nerve fibers [9, 10]. Consequently, conventional therapeutic approaches often yield suboptimal efficacy, particularly for refractory wounds that are not amenable to revascularization or remain unresponsive to standard dressing changes.

NPWT is a technique now widely applied in the care of various wound types, such as deep sternal wound infections, pressure injuries, skin grafts for chronic ulcers, and burn wounds [11, 12, 13, 14]. This technology involves the application of controlled subatmospheric pressure across a specialized wound dressing, which facilitates the removal of exudate and its collection into a canister. Through sustained topical negative pressure, NPWT effectively evacuates excess fluid, reduces tissue edema, promotes granulation tissue formation, and enhances local blood perfusion, thereby synergistically facilitating the wound healing process across multiple levels [12]. Furthermore, regarding molecular mechanisms, a meta‐analysis incorporating data from multiple animal models and clinical studies suggested that NPWT may promote healing by modulating various growth factors, such as vascular endothelial growth factor (VEGF) and fibroblast growth factor‐basic (bFGF), and inducing an anti‐inflammatory response [15].

In recent years, several RCTs have investigated the use of NPWT for DFU treatment. This study aims, through a meta‐analysis of RCTs up to October 2025, to reevaluate the impact of NPWT on DFU healing rates, amputation risk, and adverse events (AEs). Furthermore, we intend to explore sources of heterogeneity via subgroup analysis, thereby aiming to provide enhanced evidence to inform the clinical application of this technology.

## 2 Methods

### 2.1 Literature Search

A systematic search was conducted in PubMed, the Cochrane Library, Embase, and Web of Science from inception until October 2025 for randomized controlled trials (RCTs), using the main search terms “DFU” and “negative pressure therapy”. This meta‐analysis followed the Preferred Reporting Items for systematic reviews and meta‐analyses (PRISMA) criteria [16].

### 2.2 Inclusion and Exclusion Criteria

Inclusion criteria: (1) for inclusion: RCTs without language restrictions; (2) participants with a confirmed diagnosis of DFU; (3) a minimum of 10 participants completing the study in each intervention arm; (4) interventions where the experimental group received NPWT, while the control group received conventional wound care involving traditional dressings (e.g., saline gauze) or modern advanced dressings (e.g., alginate, hydrocolloid, silver‐containing dressings); and (5) studies that provided sufficient data for assessing the efficacy and safety of NPWT, such as healing rate, amputation rate, and incidence of AEs. Exclusion Criteria: (1) non‐RCTs, including case reports, case series, reviews and animal studies; (2) studies on wounds of non‐diabetic etiology, such as venous ulcers, pressure injuries, or traumatic wounds; (3) unpublished studies; (4) studies with an insufficient sample size to yield reliable results; and (5) studies with critically missing data (e.g., baseline characteristics or outcome data) that could not be obtained from the corresponding authors.

### 2.3 Literature Screening Process

During the literature screening process, two independent reviewers (HD and TJ) initially screened studies based on titles and abstracts. Articles that passed this initial screening underwent full‐text review for secondary assessment. The results were then cross‐checked, and any discrepancies were resolved through discussion between the reviewers to reach a consensus on inclusion. If necessary, a third reviewer was consulted to assist in making the final decision. In cases where information was incomplete or unclear, the original study authors were contacted for clarification. Detailed records of the reasons for inclusion or exclusion at each stage were maintained. Studies excluded after full‐text review were subsequently analyzed in the sensitivity analysis.

### 2.4 Statistical Analysis

All statistical procedures in this meta‐analysis were conducted using R software, version 4.5.1 (R Foundation for Statistical Computing). Data synthesis was carried out with the “meta” package, which was also used to generate forest plots and funnel plots. The Cochrane Risk of Bias tool (ROB 2.0) was applied to evaluate the methodological quality of the included RCTs. In light of expected clinical and methodological variations across studies, a random‐effects model was used for pooling the results. Heterogeneity was quantified with the *I* ^2^ statistic, where a value exceeding 50% was considered to represent substantial heterogeneity. Statistical significance was defined as a two‐sided *p*‐value below 0.05 for all tests. A total of 23 RCTs, providing details such as authors, publication year, sample size, participant age, and other relevant characteristics, were included in the qualitative synthesis and quantitative meta‐analysis. The primary aim was to compare the effectiveness of NPWT with conventional therapies for DFU, assessing outcomes including wound healing rate, reduction in wound area, amputation rate, and wound infection incidence. Publication bias was evaluated both visually by inspecting funnel plots and quantitatively using Egger's linear regression test. Additionally, given the observed clinical and methodological diversity among studies, subgroup analyses were performed to investigate potential sources of heterogeneity in the primary outcomes.

## 3 Results

### 3.1 Search Results and Study Characteristics

**FIGURE 1:**
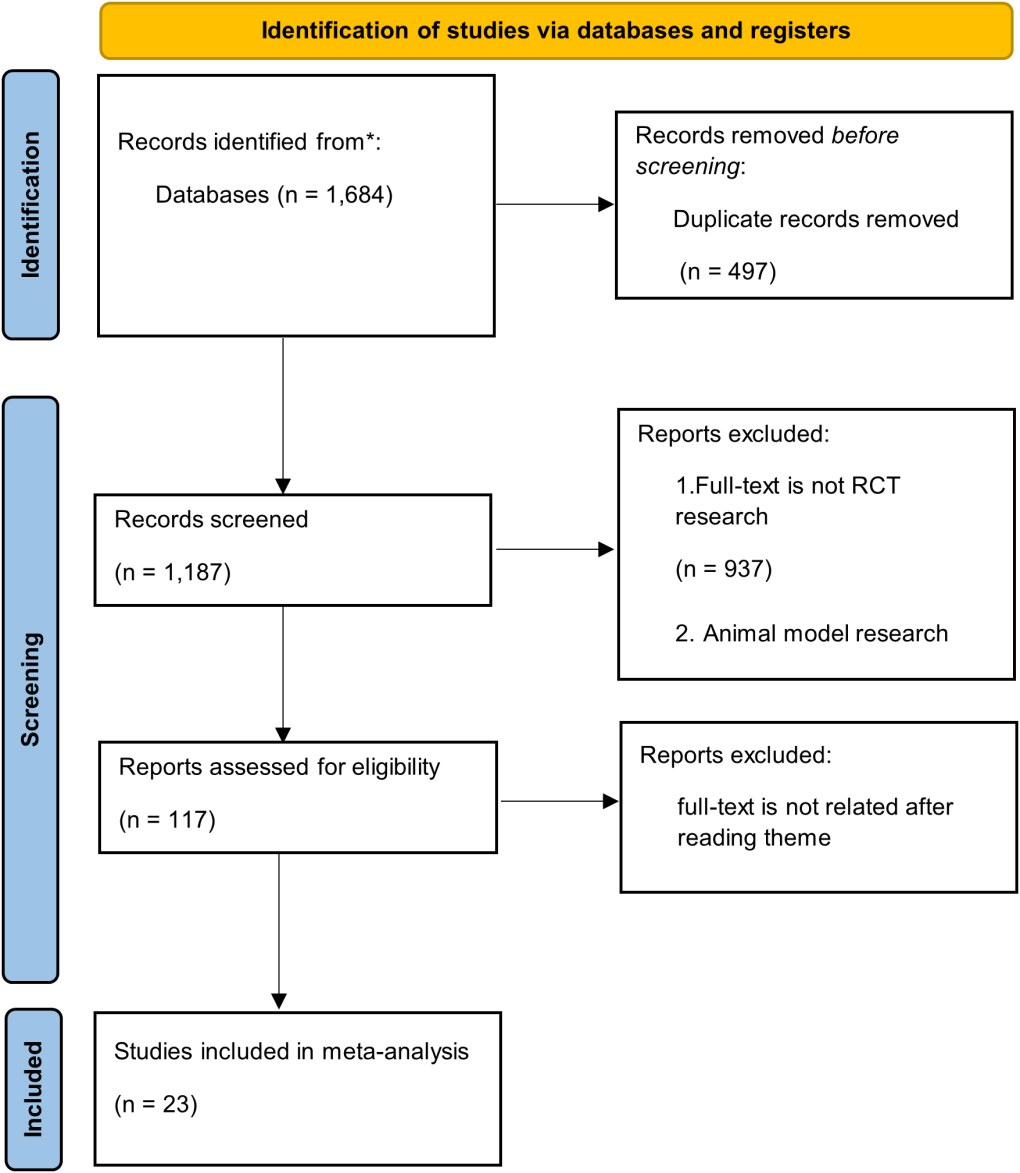
PRISMA flow diagram illustrating the study selection process.

A systematic search of the literature was conducted in PubMed, Embase, the Cochrane Library, and Web of Science. This process yielded a total of 1684 publications, which, upon consolidation of the search results, were screened for duplicates. This led to the exclusion of 498 records that were identified in more than one database. A total of 1163 articles were excluded for the following reasons: (1) 1069 articles were non‐RCTs, such as case reports, reviews, commentaries, conference abstracts, and animal experiments; (2) 86 articles were deemed irrelevant to the research topic after reading the abstracts or due to a lack of key data. Through this screening process, a total of 23 RCTs were included for meta‐analysis (Figure 1). A total of 23 clinical studies on NPWT for DFU were included in this research, with their key information summarized in Table 1 [17, 18, 19, 20, 21, 22, 23, 24, 25, 26, 27, 28, 29, 30, 31, 32, 33, 34, 35, 36, 37, 38, 39]. A publication timeline from 2005 to 2025 was covered by the included RCTs, which enrolled between 24 and 450 participants each.

**TABLE 1:** Information of the 23 included studies retrieved from PubMed, Embase, Cochrane library and web of science.

Author	Date	Country	Age	Sample size	Control group	Follow‐up duration
Armstrong, D.G	2005	USA	NPWT: 57.2 ± 13.4 CT: 60.1 ± 12.2	162	Moist wound therapy (MWT)	16 weeks
Blume, P.A	2008	USA	NPWT: 58 ± 12 CT: 59 ± 12	335	Moist wound therapy (MWT)	16 weeks
Sepúlveda, G	2009	Chile	NPWT: 61.5 ± 10 CT: 62.1 ± 8	24	Moist wound therapy (MWT)	6 weeks
Galstyan, G.R	2016	Russia	NPWT: 60.0 ± 8.9 CT: 60.0 ± 11.1	42	Standard wound care	9 days
Karam, R.A	2018	Saudi Arabia	NPWT: 58.2 ± 7.1 CT: 59.1 ± 7.2	40	Moist wound therapy (MWT)	6 weeks
Bin Saeed, M	2019	Pakistan	NPWT: 53.53 ± 3.80 CT: 55.21 ± 5.76	64	Moist Dressing	2 weeks
Seidel, D	2020	Germany	NPWT: 67.6 ± 12.3 CT: 68.1 ± 11.5	345	Moist wound therapy (MWT)	16 weeks
Malekpour Alamdari, N	2021	Iran	NPWT: 70.31 ± 5.92 CT: 71.80 ± 6.32	60	Silver sulfadiazine dressing	12 weeks
Maranna, H	2021	India	NPWT: 50.23 ± 10.52 CT: 49.00 ± 10.14	45	Conventional saline dressing	21 weeks
Anjum, W	2022	Pakistan	NPWT: 42.95 ± 9.29 CT: 46.30 ± 9.33	40	Conventional wound dressing	2 weeks
Srivastava, V	2022	India	NPWT: 37.32 ± 6.64 CT: 36.74 ± 7.22	55	Conventional wound dressing	3 weeks
Dong, B	2023	China	NPWT: 66.72 ± 10.09 CT: 70.76 ± 11.13	50	Conventional wound dressing	1 year
Galal, A.M.M	2023	Egypt	NPWT: 53.47 ± 11.01 CT: 49.53 ± 7.28	30	Tetra‐silver nitrate dressing	6 weeks
Ali, R.Z	2024	Pakistan	NPWT: 58.4 ± 10.3 CT: 58.8 ± 10.1	92	Standard wound care	6 weeks
Kamalakkhannan, C	2024	India	NPWT: 59.42 ± 6.18 CT: 61.86 ± 8.56	80	Conventional wound dressing	—
Mahmoud, A.A.H.	2024	Egypt	NPWT: 63.30 ± 8.36 CT: 61.35 ± 7.56	40	Nano‐silver dressing	6 weeks
Manoop, B	2024	India	NPWT: 43 ± 12.3 CT: 45.5 ± 10.9	70	Conventional wound dressing	6 days
Mehta, V.P.	2024	India	12–84	100	Conventional wound dressing	4 weeks
Patel, T.B	2024	India	18–70	60	Standard wound care	12 weeks
Yadav, O.P	2024	India	NPWT: 56.33 ± 1.23 CT: 56.23 ± 1.42	55	Conventional wound dressing	3 weeks
Gu, H	2025	China	60 ± 10	450	Moist wound therapy (MWT)	9 months
Ranjan, P	2025	India	< 70	50	Conventional wound dressing	8 weeks
Suman, A., V	2025	India	NPWT: 52.20 ± 9.50 CT: 53.78 ± 10.08	52	Conventional wound dressing	4 weeks

### 3.2 Risk Assessment

**FIGURE 2:**
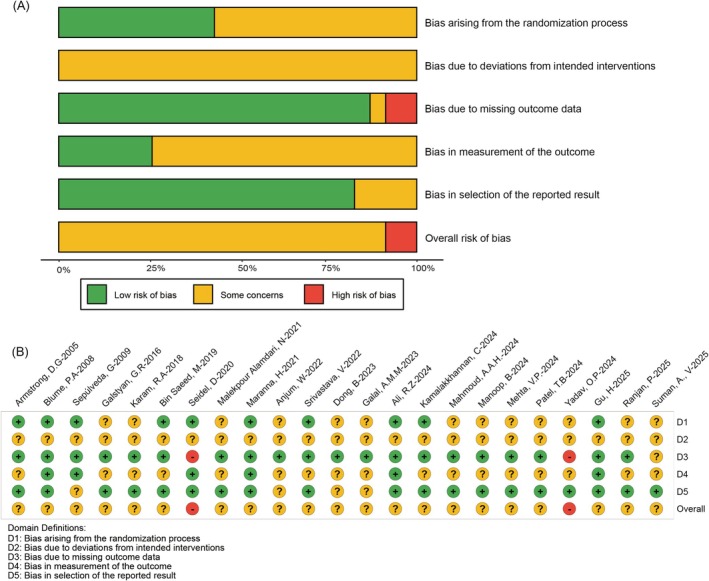
Risk of bias assessment. (A) Traffic light plot of domain‐specific risk of bias judgments. (B) Summary plot of risk of bias across all included studies.

An evaluation of the methodological quality of the included RCTs was conducted with the revised Cochrane Risk of Bias tool (ROB 2.0). The assessment, summarized in Figure 2, covers five critical bias domains. A prevalent limitation was identified in the randomization process; although sequence generation was typically reported, inadequate details on allocation concealment in partial trials conferred a rating of “some concerns.” Similarly, the inability to blind personnel and participants to the NPWT intervention resulted in a consistent rating of “some concerns” for deviations from intended interventions. Acknowledging these potential biases is crucial for the contextual interpretation of the meta‐analysis findings. It is important to emphasize that the identified risks of bias may potentially influence the estimation of treatment effects. By applying the ROB 2 tool in this detailed manner, our analysis not only enhances the transparency of the meta‐analytic findings but also provides essential methodological context for interpreting the results, thereby supporting the derivation of more robust clinical conclusions.

### 3.3 Wound Healing Rate

**FIGURE 3:**
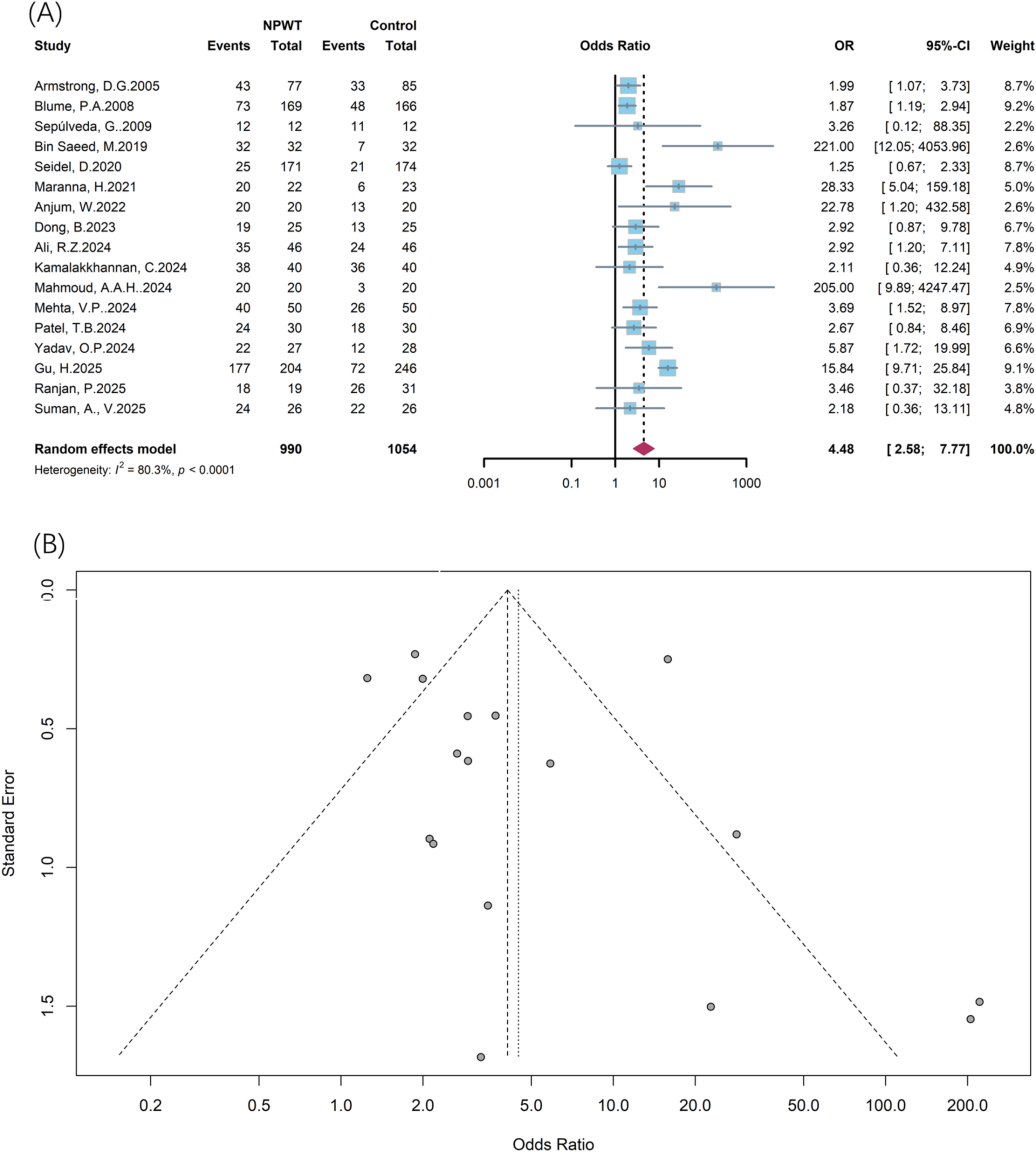
(A) Forest plot for the comparison of wound healing rate between NPWT and conventional wound care. (B) Funnel plot for the assessment of potential publication bias.

A meta‐analysis of 17 RCTs showed a significantly benefit of NPWT in promoting wound healing. The OR was 4.48 (95% CI: 2.58–7.77), indicating that NPWT significantly increased the healing rate compared to conventional wound therapy. The forest plot displays the individual study estimates and their respective weights in the meta‐analysis, revealing a consistent direction of effect favoring NPWT across the majority of studies, despite variations in the magnitude of the effect size (Figure 3A). The observation of pronounced heterogeneity (*I* ^2^ = 80.3%, *p* < 0.0001) among the included studies warranted the application of a random‐effects model for pooling the results. The stability of the result was tested by sensitivity analysis, which indicated that the overall finding regarding the treatment effect was not substantially altered by omitting any single study. To evaluate potential publication bias, a funnel plot was generated and visually examined (Figure 3B). The observed asymmetry could be speculated to stem from clinical heterogeneity or the varying precision of the included studies. To quantitatively assess the potential for publication bias in the studies reporting wound healing rates, Egger's linear regression test was performed. The test revealed no statistically significant evidence of funnel plot asymmetry (*t* = 1.04, df = 15, *p* = 0.314).

### 3.4 Subgroup Analysis

To explore the substantial heterogeneity (*I* ^2^ = 80.3%) observed for the wound healing rate outcome with NPWT in DFU, we conducted subgroup analyses based on pre‐specified clinically relevant variables.

#### 3.4.1 The Type of Control Dressing

**FIGURE 4:**
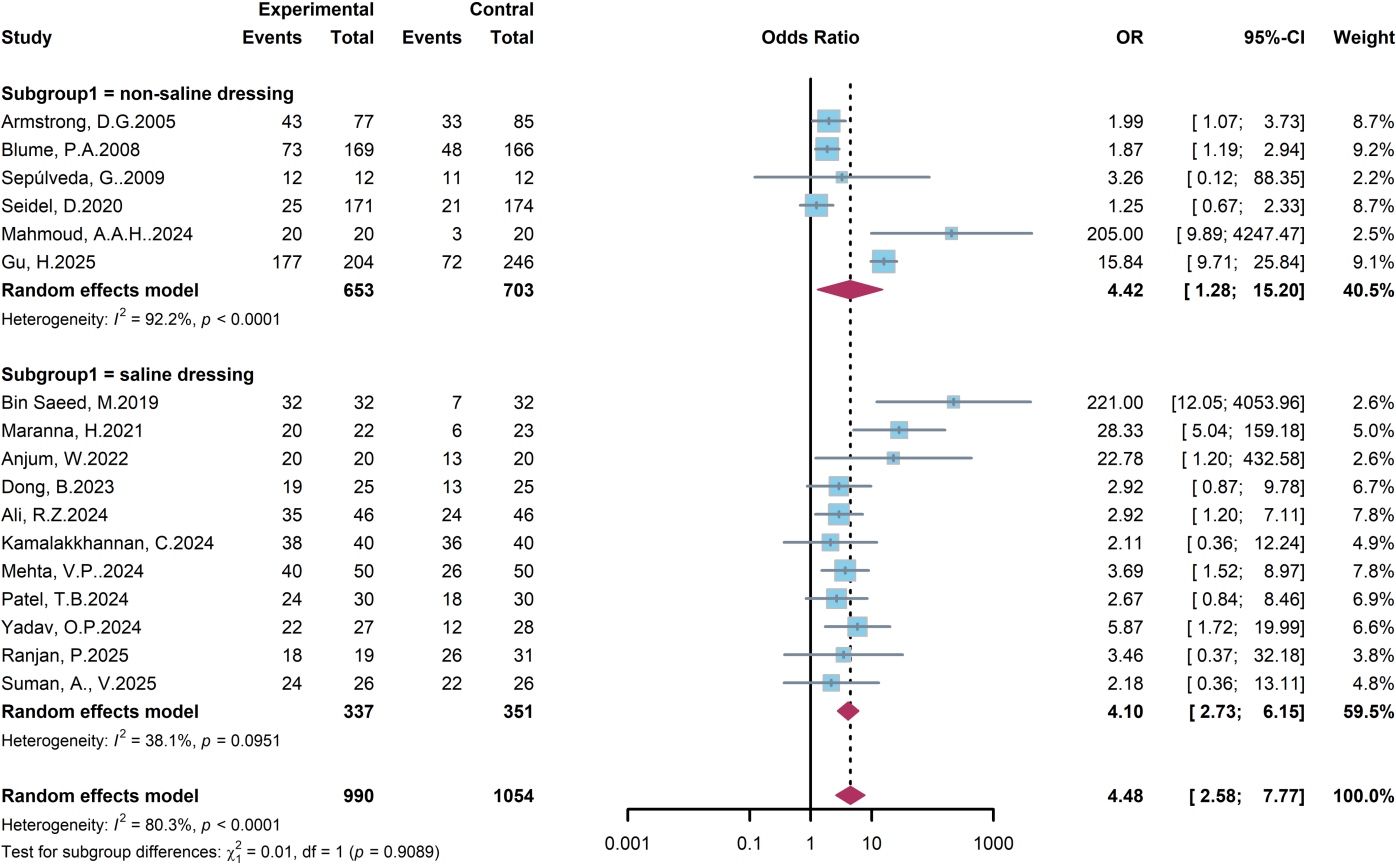
Forest plot of subgroup analysis for wound healing rate based on the type of control dressing.

To evaluate the impact of NPWT on wound healing in patients with DFU, a meta‐analysis including 17 RCTs was performed (Figure 4), with subgroups stratified by the type of dressing used (non‐saline vs. saline). Subgroup analyses revealed consistent benefits in both the non‐saline dressing group (OR = 4.42, 95% CI: 1.28–15.20) and the saline dressing group (OR = 4.10, 95% CI: 2.73–6.15), with no significant difference between subgroups (*p* = 0.9089). Heterogeneity was notably higher in the non‐saline subgroup (*I* ^2^ = 92.2%) than in the saline subgroup (*I* ^2^ = 38.1%). In summary, NPWT significantly enhances the probability of wound healing in DFU patients, irrespective of the dressing type used alongside the therapy.

#### 3.4.2 Follow‐Up Duration

**FIGURE 5:**
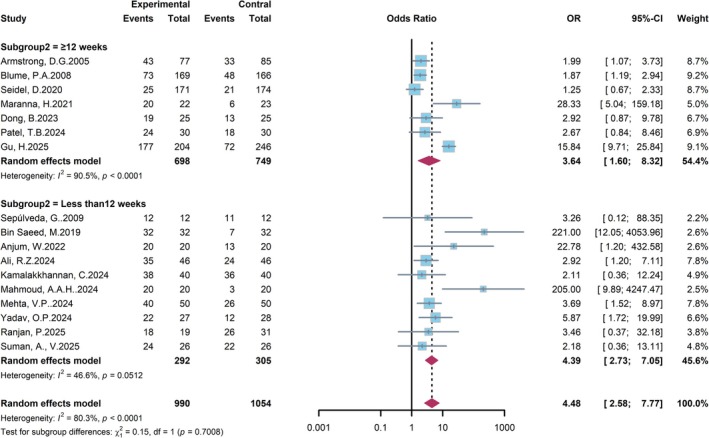
Forest plot of subgroup analysis by follow‐up duration.

A subgroup analysis stratified by follow‐up duration was performed to evaluate the effect of NPWT on wound healing in DFU patients (Figure 5). In the subgroup with longer follow‐up (≥ 12 weeks), NPWT remained associated with a significantly higher healing rate (OR = 3.64, 95% CI: 1.60–8.32), albeit with considerable heterogeneity (*I* ^2^ = 90.5%). Similarly, in the subgroup with shorter follow‐up (< 12 weeks), a significant treatment effect was also observed (OR = 4.39, 95% CI: 2.73–7.05), with moderate heterogeneity (*I* ^2^ = 46.6%). The result for subgroup differences indicated no statistically significant interaction between follow‐up duration and treatment effect (*p* = 0.7008). In conclusion, NPWT significantly improves wound healing rates irrespective of the follow‐up duration.

#### 3.4.3 Patient Age

**FIGURE 6:**
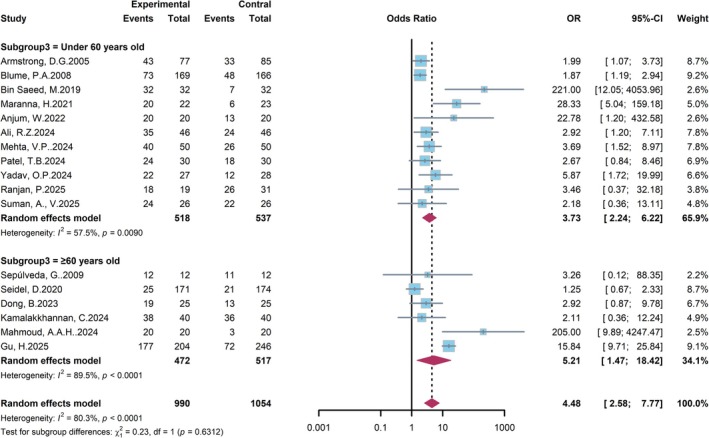
Forest plot of subgroup analysis by patient age.

We further conducted a subgroup analysis categorized by patient age to explore its potential influence on the therapy effect of NPWT (Figure 6). In the subgroup of patients under 60 years old, NPWT was associated with a significantly higher likelihood of wound healing (OR = 3.73, 95% CI: 2.24–6.22), with moderate heterogeneity (*I* ^2^ = 57.5%). A similarly pronounced treatment effect was observed in the subgroup of patients aged 60 years or older (OR = 4.84, 95% CI: 1.79–13.09), although the evidence in this cohort was characterized by considerable heterogeneity (*I* ^2^ = 89.5%). The result of subgroup differences revealed no statistically significant interaction between patient age and the treatment effect (*p* = 0.6312), indicating that the superiority of NPWT was consistent across the two age groups.

#### 3.4.4 Ulcer Severity

**FIGURE 7:**
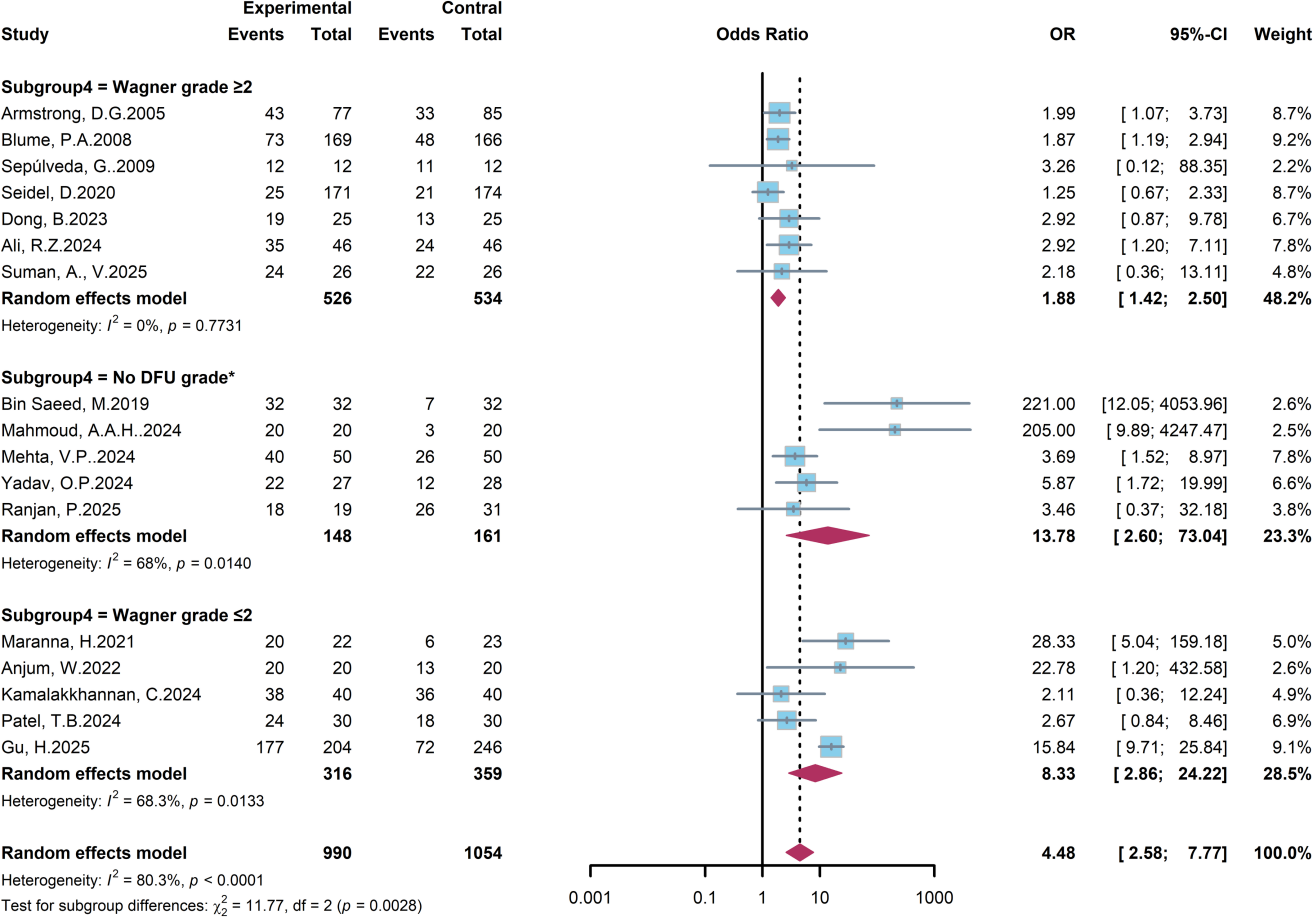
Forest plot of subgroup analysis by ulcer severity (Wagner grade).

We also performed a subgroup analysis based on the baseline ulcer severity according to the Wagner grade to evaluate its impact on the treatment effect of NPWT (Figure 7). Statistically significant differences in the treatment effect were observed across the severity subgroups (p for subgroup differences = 0.0028). In the subgroup with the most severe ulcers (Wagner grade ≥ 2), the OR was 1.88 (95% CI: 1.42–2.50), indicating a beneficial effect of NPWT with negligible heterogeneity (*I* ^2^ = 0%). In contrast, the point estimate for the treatment effect was substantially higher in the subgroup with less severe ulcers (Wagner grade ≤ 2), with an OR of 8.33 (95% CI: 3.86–24.22). This differential effect may be attributed to the greater intrinsic healing potential and fewer comorbidities in patients with less severe ulcers. These results suggest that while NPWT is effective across all severity levels, the magnitude of its relative benefit is most pronounced in patients with less severe DFU.

### 3.5 Adverse Events

**FIGURE 8:**
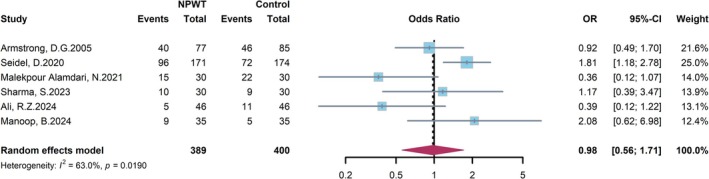
Forest plot comparing the risk of total adverse events between NPWT and conventional wound care.

The analysis for AEs encompassed six RCTs comparing NPWT to control in DFU patients (Figure 8). According to the random‐effects model, the incidence of AEs did not differ significantly between the intervention and control groups. This was evidenced by an OR of 0.98 (95% CI: 0.56–1.71). A moderate level of heterogeneity was noted among these studies (*I* ^2^ = 63.0%, *p* = 0.019). The point estimates of most individual studies were distributed around the null effect line (OR = 1), indicating no clear superiority of NPWT over conventional therapy in terms of AE risk. In conclusion, this meta‐analysis suggests that NPWT does not significantly increase or decrease the risk of AEs compared to conventional wound therapy in DFU patients.

### 3.6 Amputation

**FIGURE 9:**
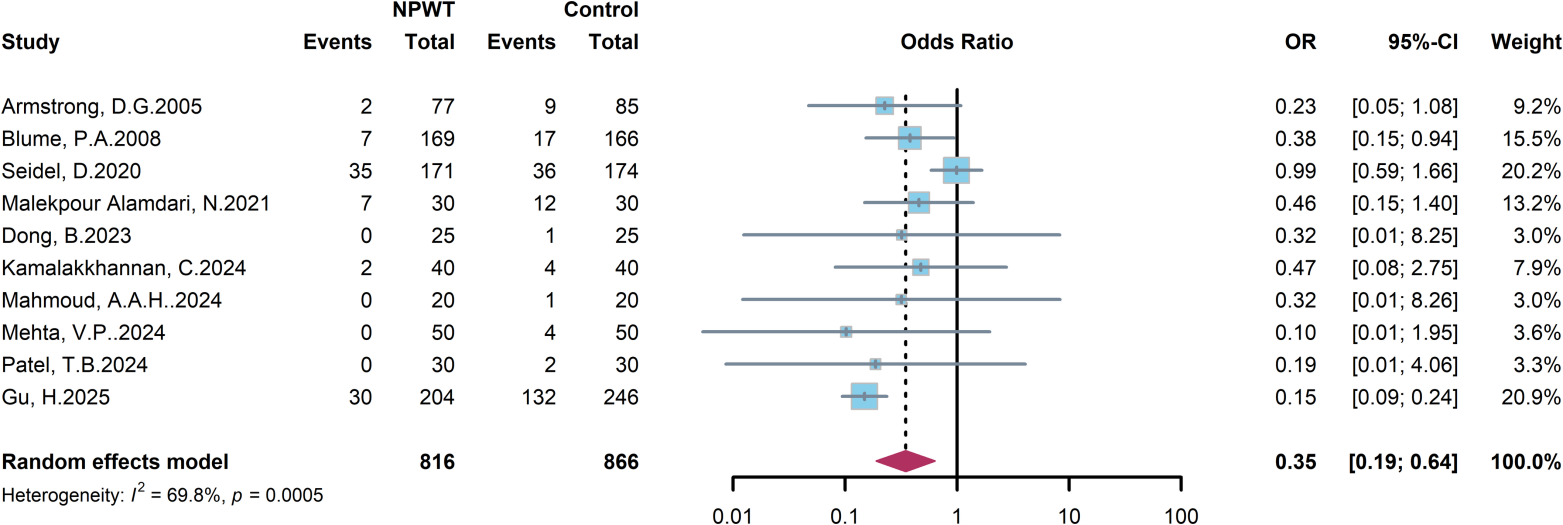
Forest plot comparing the risk of amputation between NPWT and conventional wound care.

10 RCTs were included to compare the effect of NPWT versus conventional wound therapy on reducing the risk of amputation in patients with DFU (Figure 9). A finding from the random‐effects model meta‐analysis yielded an OR of 0.35 (95% CI: 0.19–0.64), confirming a statistically significant advantage for NPWT over the control group in reducing amputation risk. This represents an approximately 65% reduction in amputation risk associated with NPWT for DFU patients. Although moderate heterogeneity was observed across the studies (*I* ^2^ = 69.8%, *p* = 0.0005), the point estimates of the effect size for the majority of individual studies lay to the left of the line of null effect (OR = 1). In summary, this meta‐analysis confirms that NPWT is a significantly more effective treatment strategy than conventional wound therapy for preventing amputation in patients with DFU.

### 3.7 Ulcer Area

**FIGURE 10:**
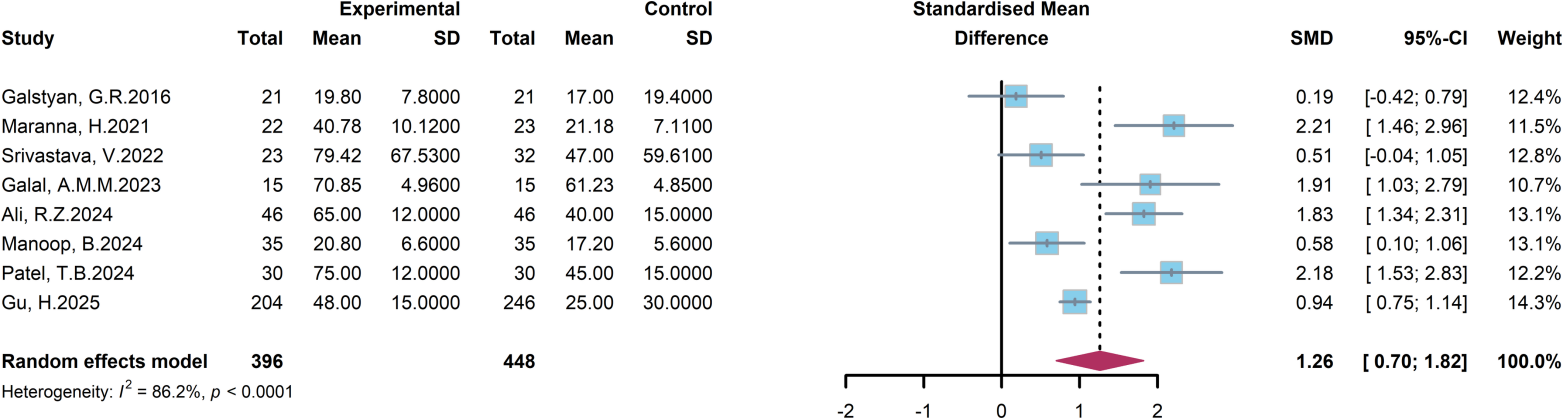
Forest plot comparing the reduction of ulcer area between NPWT and conventional wound care in the patients with DFU.

A meta‐analysis of eight studies evaluating the efficacy of NPWT versus conventional treatment in reducing the wound area of DFU was performed (Figure 10). The standardized mean difference (SMD) was pooled using a random‐effects model. The pooled result indicated that NPWT was significantly superior to the control group in reducing wound area, with a combined SMD of 1.26 (95% CI: 0.70–1.82), and the difference was significant. Despite the presence of high heterogeneity among the included studies (*I* ^2^ = 86.2%, *p* < 0.0001), the direction of effect was consistent across all individual studies, uniformly supporting the therapeutic efficacy of NPWT for DFU.

## 4 Discussion

This updated meta‐analysis, incorporating evidence from 23 (RCTs), unequivocally demonstrates that NPWT confers significant advantages over conventional wound care in the management of DFU. Our findings confirm that NPWT not only accelerates wound healing and reduces ulcer area but also, most importantly, dramatically reduces the risk of major amputation by approximately 65%, without increasing the overall burden of AEs.

The pronounced beneficial effect of NPWT on DFU healing (OR = 4.48) can be attributed to its multifaceted mechanisms of action. Firstly, appropriate negative pressure levels and duration have been shown to enhance local blood perfusion in the wound bed, as evidenced by numerous studies in animal models [40, 41, 42] and humans [43, 44, 45]. Ma Z et al. further reported that NPWT promotes the overexpression of Angiopoietin‐1, Tie‐2, *α*‐SMA, and Collagen IV, thereby facilitating wound perfusion and microvascular maturation [41]. Concurrently with improved perfusion, the negative pressure environment and connected drainage system effectively remove inflammatory exudate, which also contributes to the healing process. Secondly, NPWT promotes the formation of granulation tissue. Dunn et al., in a study assessing wounds in 131 patients (including traumatic, postoperative, and chronic wounds), observed a significant increase in red granulation tissue (*p* = 0.007) [46]. Similarly, Lee HJ et al. demonstrated that NPWT facilitates the rapid formation of healthy granulation tissue in acute traumatic wounds [47]. Furthermore, Huang et al. discovered that NPWT promotes DFU healing by downregulating the expression of miR‐155 [48]. Additionally, a growing body of research indicates that NPWT can upregulate the expression of various healing‐related factors. Cao et al. found that NPWT enhances VEGF expression in wound vascular endothelial cells and fibroblasts (*p* < 0.05) [49]. Labler L et al. reported that NPWT accelerates granulation tissue formation by modulating the expression of IL‐6, IL‐8, and VEGF [50]. McNulty et al. observed that NPWT significantly increases levels of Transforming Growth Factor‐*β* and Platelet‐Derived Growth Factor (*α* and *β* isoforms) by 80% and 53%, respectively (*p* < 0.05) [51]. These pro‐healing factors play crucial roles in the wound healing cascade.

Although this meta‐analysis found that the NPWT group did not increase the occurrence of AEs compared to the control group, vigilance regarding these events remains necessary. AEs associated with NPWT primarily include pain, bleeding, and infection. Treatment‐related pain, in particular, can negatively impact patient compliance and consequently treatment efficacy [52].

Despite performing multiple subgroup analyses, the sources of heterogeneity for the primary outcomes remain unclear. Firstly, the included studies generally lacked detailed reporting of specific NPWT technical parameters—such as negative pressure intensity, pressure application mode (continuous vs. intermittent), and wound interface type (e.g., polyurethane foam vs. gauze). These variables are likely significant contributors to the substantial statistical heterogeneity (*I* ^2^ = 80.3%) observed in our primary outcome analysis. Future studies are needed to clarify the impact of these factors on NPWT treatment efficacy, thereby guiding clinicians in developing personalized NPWT regimens for individual patients with DFUs. Secondly, due to incomplete reporting of baseline clinical characteristics—such as glycemic control levels, diabetes duration, and history of diabetic foot—subgroup analyses based on these factors were not performed.

This study has several limitations. First, due to the inherent nature of the NPWT intervention, blinding of patients and healthcare providers was challenging in the included trials, potentially introducing performance and detection bias. Second, the substantial heterogeneity observed (*I* ^2^ = 80.3%) largely remained unexplained by the subgroup variables analyzed. Third, despite a comprehensive literature search, the asymmetry suggested by the funnel plot indicates that publication bias cannot be entirely ruled out. Fourth, the generally small sample sizes observed in many of the included RCTs constitute a notable limitation, thereby calling for future trials that are both larger in scale and methodologically sound.

## 5 Conclusion

Our meta‐analysis provides evidence that NPWT can significantly promote wound healing, reduce amputation, and does not increase the occurrence of AEs. The results support the role of NPWT as a first‐line adjuvant treatment. Future research should focus on personalized application and long‐term efficacy.

## Author Contributions

**Hai Du** and **Tao Jiang** performed the literature investigation and drafted the manuscript. **Zhenbing Chen** and **Xiaofan Yang** conceived the project and revised the manuscript. All authors read and approved the final manuscript. **Hai Du:** writing – review and editing, writing – original draft, Investigation. **Tao Jiang:** writing – review and editing, investigation. **Xiaofan Yang:** supervision, funding acquisition. **Zhenbing Chen:** supervision, funding acquisition.

## Funding

This work was supported by the National Natural Science Foundation of China (grant number 82472566, 82370838), the Health Science and Technology Project of Hubei Province (grant number WJ2025Z007), the Key Research and Development Program of Hubei Province (grant number 2024BCB039), and the Medical Science and Technology Research Foundation of Guangdong Province (grant number B2024010).

## Conflicts of Interest

The authors declare no conflicts of interest.

## Data Availability

The data that support the findings of this study are available from the corresponding author upon reasonable request.
